# Acute kidney injury is associated with decreased platelet function in domestic cats

**DOI:** 10.1093/jvimsj/aalag115

**Published:** 2026-06-10

**Authors:** Matthew R Kornya, Shauna L Blois, Anthony C G Abrams-Ogg

**Affiliations:** Department of Clinical Studies, Ontario Veterinary College, University of Guelph, Guelph, ON, Canada; Department of Clinical Studies, Ontario Veterinary College, University of Guelph, Guelph, ON, Canada; Department of Clinical Studies, Ontario Veterinary College, University of Guelph, Guelph, ON, Canada

**Keywords:** aggregometry, azotemia, kidney injury, platelet aggregation

## Abstract

**Background:**

Acute kidney injury (AKI) is associated with platelet dysfunction in several species, but has been minimally investigated in cats. The Platelet Function Analyzer-200 (PFA-200) and Multiplate® (MP) investigate platelet function using different mechanisms.

**Hypothesis/Objectives:**

Cats with AKI will show evidence of platelet dysfunction on PFA-200 collagen/adenosine diphosphate (Col/ADP) and MP ADP, Col, and arachidonic acid (ASPI) assays.

**Animals:**

Fifteen cats presented to a tertiary referral hospital with a diagnosis of AKI and 15 control cats.

**Methods:**

Complete blood count, serum biochemistry panel, PFA-200 Col/ADP, and MP ADP, Col, and ASPI assays were analyzed for associations between platelet count and function and AKI status and cause of AKI. Association between severity of platelet dysfunction and severity of azotemia also was assessed.

**Results:**

Cats with AKI had higher median PFA-200 Col/ADP closure time (CT) than control cats (109 s vs 56 s; *P* = .001) and lower median MP area under curve (AUC) in response to ADP (62 vs 108; *P* = .002), Col (60 vs 98; *P* = .007), and ASPI (22 vs 46; *P* = .001). No difference was found between cats with ureteral obstruction vs other causes of AKI in median MP ADP AUC (*P* = .78); MP ASPI AUC (*P* = .31), or MP Col AUC (*P* = .18), but obstructed cats had higher median PFA-200 CT (154 s vs 68 s; *P* = .02). Among cats with AKI, no association was found between serum urea, creatinine, or phosphorus concentrations, or urine specific gravity, and any platelet parameter.

**Conclusions and clinical importance:**

Cats with AKI have evidence of platelet dysfunction. Although the clinical relevance of this finding is unknown, it should be to considered when undertaking invasive procedures.

## Introduction

Azotemia has been associated with thrombocytopathy in both human and veterinary medicine.^1–5^ Classically, azotemia has been considered a hypocoagulable state, but increasing evidence suggests that kidney disease causes a complex mixed hemostatic disturbance, and uremic patients may have both hemorrhagic and thrombotic tendencies.^6^ Azotemia-associated platelet dysfunction may contribute to post-operative bleeding, bleeding after sampling procedures (eg, renal aspirates, renal biopsies), or to gastrointestinal blood loss.

Disturbance of primary hemostasis is most commonly implicated in uremic bleeding as a consequence of changes in platelet number or function.^1^^,^^7^^,^^8^ Humans with kidney disease have decreased platelet aggregation, and alterations in von-Willebrand’s factor (vWF) concentrations or binding affinity may play a role in this change.^2^ However, patients with renal disease often show signs of glomerular thrombosis and changes associated with platelet activation.^6^^,^^9^

In veterinary medicine, disorders of primary hemostasis associated with renal dysfunction have been less well defined. According to one report, dogs with uremia may develop a type-II von-Willebrand disease phenotype, resulting in platelet function defects.^4^ However, a surgical model of acute kidney injury (AKI) in dogs found normal or increased vWF concentrations and no difference in multimer distribution, suggesting this abnormality may not be universal.^10^ Another experimental model showed that platelet count remained normal whereas buccal mucosal bleeding time and glass bead retention showed changes consistent with thrombopathy.^11^

Some data suggest that the acuity of kidney injury may affect coagulation status, with hypocoagulability in dogs with AKI and hypercoagulability in dogs with chronic kidney disease (CKD).^1^^,^^11^^,^^12^

Minimal information is available on the effects of AKI on platelet function in cats. The only report to investigate platelet function in azotemic cats recruited a small number of cats with AKI, and found evidence of platelet hyper-reactivity.^13^ This unexpected finding conflicts with data in other species, as well as the anecdotal impressions of many veterinarians. Understanding if cats with kidney injury are more likely to have bleeding or thrombotic events is important to their management and may help in planning interventions, measuring risk, and informing treatment. Cats with platelet hyperfunction may benefit from antithrombotic treatment, whereas those with hypofunction may require closer monitoring for bleeding risk, and possibly interventions to decrease the risk of hemorrhage. As such, more information on platelet function in cats in the context of AKI is needed.

Multiple electrode aggregometry (MEA) is an impedance-based method of platelet analysis that utilizes samples of whole blood exposed to an agonist while warmed and under constant agitation as a clot forms on electrodes.^14^ The Multiplate^®^ analyzer (MP; Roche Diagnostics, Rotkreuz, Switzerland) is a benchtop MEA analyzer that runs assays in duplicate in a single test cell and reports platelet function as “Units” (U) of area under the curve (AUC).^15^^,^^16^

The Platelet Function Analyzer 200 (PFA-200; Siemens Healthcare Diagnostics, Marburg, Germany)^17^ assesses the ability of platelets to adhere to a perforated membrane coated in agonists under high-shear conditions,^18^ allowing it to detect platelet–platelet interactions and interactions with a membrane. Several test cartridges are available for the PFA, that assess platelet function using different agonists including adenosine diphosphate and collagen (ADP/Col).

The purpose of our study was to investigate the effects of AKI on platelet function in cats using MP with ADP, arachidonic acid (ASPI), and Col agonists; and using the PFA-200 with ADP/Col. Our hypothesis was that cats with AKI would exhibit decreased platelet function compared with controls.

## Materials and methods

Ethical approval for the study was obtained from the university animal care committee. See Appendix for inclusion and exclusion criteria, and criteria for categorization.

From each cat in both control and AKI groups, 1.8 mL of blood was collected and transferred to a 3.2% sodium citrate tube (Becton Dickinson, Mississauga, ON, Canada). Ease of venipuncture and consistency of blood flow were scored on a 3 point scale using a previously described system to evaluate redirection and flow stoppage, and the sample excluded if phlebotomy was scored as a “3.”^19–21^

Control cats were recruited on a volunteer basis from students, faculty, and staff. They were confirmed to be healthy based on medical history, physical examination, and routine blood testing (CBC, serum biochemistry profile, FeLV/FIV testing). Control group cats were excluded if they were thrombocytopenic or anemic. Cats were gently manually restrained and jugular venipuncture performed using a 22 g 1″ needle attached to a 6 mL syringe. Blood (1.8 mL) was collected and transferred to a 3.2% sodium citrate tube (Becton Dickinson, Mississauga, ON, Canada) for coagulation testing, as well as a sample in EDTA for CBC and heparin for biochemical analysis. See Appendix for details of laboratory analysis.

Statistical analysis was performed using DataTab (DATAtab e.U. Graz, Austria). Data were assessed for normality using the Shapiro–Wilk test and inspection of Q–Q plots. Differences in platelet function and count between control and AKI cats and subgroup analysis were investigated using the T-test or Mann–Whitney U-test, depending on data normality. Associations between platelet parameters and creatinine, urea, and phosphorus concentrations, and specific gravity were investigated using Pearson correlation coefficients. Associations between age, sex, and platelet function tests were evaluated using Pearson correlation analysis and T-tests.

A power analysis was performed using previously published data on feline platelet function and azotemia as a baseline.^13^ This analysis suggested a sample size of 10 cats per group would be sufficient to give 80% power at an alpha of 0.05 with an effect size of 0.5 using Cohen’s d. A planned interim power analysis after recruiting 8 cats per group suggested 15 cats would be required to achieve this power level and effect size with all agonists on both analyzers, and thus recruitment was continued to include 15 cats per group. Doing so ultimately resulted in a Cohen’s d of 1.98. This sample size did not ensure adequate power for subgroup analyses.

## Results

Fifteen cats were enrolled in each group. The control group had a median age of 6 years (range, 1-12 years), and the AKI group a median age of 8 years (range, 2-13 years). The control group had 7 spayed females and 8 neutered males; the AKI group had 6 spayed females and 9 neutered males. No difference was found in median age (*P* = .06) or sex (*P* = .77) between groups. The control group had 9 domestic shorthair cats, 4 domestic longhair, 1 Siberian Forest Cat, and 1 Russian Blue. The AKI group had 9 domestic shorthair cats, 2 domestic longhair, and 1 each of Bombay, Persian, Siamese, and Ragdoll. All samples were collected within 6 h of admission.

No association was found between age and MP ADP AUC (*P* = .54), MP ASPI AUC (.11), MP Col AUC (*P* = .13), or PFA-200 Col/ADP CT (*P* = .20). No association was found between sex and MP ADP AUC (*P* = .51), MP ASPI AUC (*P* = .45), MP Col AUC (*P* = .76), or PFA-200 Col/ADP CT (*P* = .14).

Considering only control cats, no association was found between MP ADP AUC (*P* = .57), MP ASPI AUC (*P* = .79), MP Col AUC (*P* = .29), or PFA-200 Col/ADP CT (*P* = .19) and platelet count or between MP ADP AUC (*P* = .97), MP ASPI AUC (*P* = .50), MP Col AUC (*P* = .21), or PFA-200 Col/ADP CT (*P* = .51) and hematocrit.

Considering only AKI cats, no association was found between MP ADP AUC (*P* = 0.40), MP ASPI AUC (*P* = 0.79), MP Col AUC (*P* = .05), or PFA-200 Col/ADP CT (*P* = .81) and platelet count or between MP ADP AUC (*P* = .41), MP ASPI AUC (*P* = .50), MP Col AUC (*P* = .33), or PFA-200 Col/ADP CT (*P* = .92) and hematocrit. The cause of AKI was ureteral obstruction in 9 cats, pyelonephritis in 3 cats, and unknown cause in 3 cats. No cat with ureteral obstruction had a positive pyelocentesis culture.

Of the 9 cats diagnosed with ureteral obstruction, 7 were diagnosed based on ultrasound findings alone. These cats had a median renal pelvic diameter of 13.5 mm (range, 7.2-19.9 mm); 5 of these cats had ureteral calculi identified, and the other 2 had focal ureteral narrowing. The other 2 cats required pyelography to reach a diagnoses; they had a median renal pelvic diameter of 5.3 mm (range, 3.8-6.7 mm).

For subgroup analysis, cats were grouped as having “obstructive” or “non-obstructive” causes of AKI.

No difference was found in median platelet count (AKI, 249 × 10^9^/L; range, 147 × 10^9^/L-467 × 10^9^/L; control, 323 × 10^9^/L; range, 162 × 10^9^/L-555 × 10^9^/L; *P* = .06), mean platelet volume (AKI, 17.56 ± 2.9 fL; control, 15.53 ± 3.15 fL *P* = .08), or mean plateletcrit (AKI, 0.41+/−0.14%; control, 0.48+/−0.12%; *P* = .39) between groups. Median hematocrit was significantly lower in the AKI group (AKI median, 29%; range, 21%-44% vs control median, 36%; range, 30%-47%; *P* = .01). Two samples in the AKI group and one sample in the control group were excluded from analysis because of moderate to marked platelet clumping. Of the included samples, 11 of the AKI and 10 of the controls showed no to minimal clumping, and 4 of the AKI and 5 of the controls showed mild clumping.

The mean serum creatinine concentration in cats with AKI was 692 +/- 518.74 μmol/L and the median urea concentration was 38.5 mmol/L (range, 12.8-89 mmol/L). Two cats had International Renal Interest Society (IRIS) grade II AKI, 4 cats had IRIS grade III AKI; 6 cats had IRIS grade IV AKI, and 3 cats had IRIS grade V AKI. The median serum creatinine concentration in control cats was 99 μmol/L (range, 70-144 μmol/L) and the median serum urea concentration was 8.3 mmol/L (range, 6.3-12.4 mmol/L).

**Figure 1:**
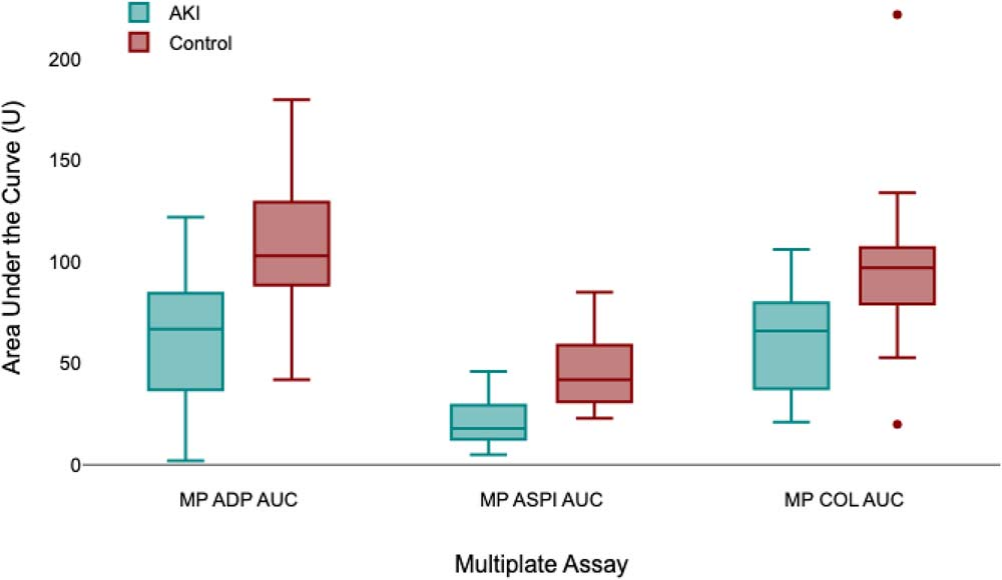
Area under the curve (AUC) of multiple electrode impedance aggregometry measured in aggregation units (U) using adenosine diphosphate (ADP), arachidonic acid (ASPI) and collagen (Col) as reagents; in cats with AKI and control cats. Solid line: median; box: quartiles; whiskers: minimum and maximum; dots: outliers.

**Figure 2:**
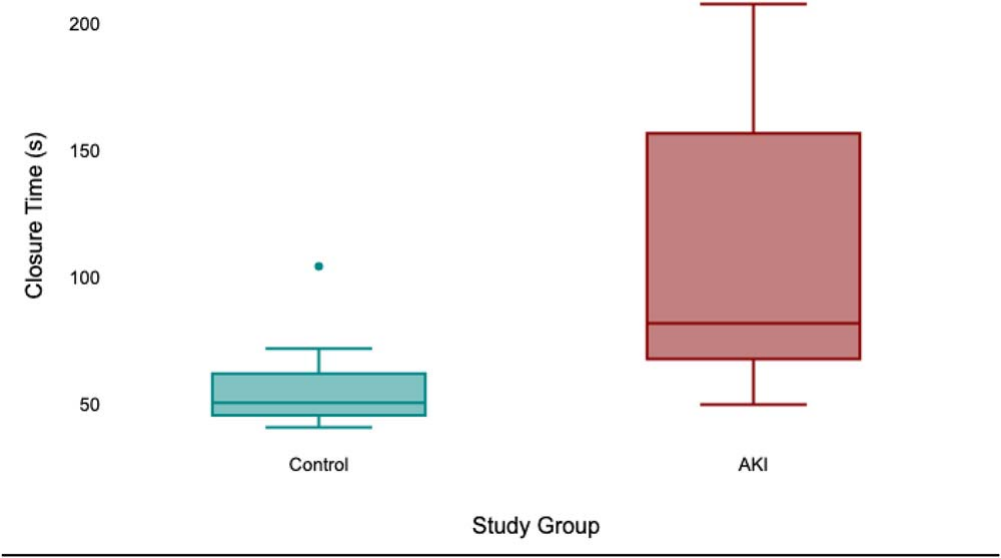
Closure time (CT) of Platelet Function Analyzer-200 Col/ADP cartridges in cats with AKI and control cats. Solid line: median; box: quartiles; whiskers: minimum and maximum; dots: outliers.

Cats with AKI had significantly higher mean PFA-200 Col/ADP CT than control cats (109 s vs 56 s; *P* < .001; Figure 1). Cats with AKI had a significantly lower median aggregation on MP analysis in response to ADP (62 U vs 108 U; *P* = .002), Col (60 U vs 98 U; *P* = .01), and ASPI (22 U vs 46 U; *P* = .001; Figure 2; Table 1). When assessing platelet function based on cause of AKI, no difference was found between cats with ureteral obstruction vs other causes on MP ADP AUC (median, 67 U vs 64 U; *P* = .78); MP ASPI AUC (median, 17 U vs 25 U; *P* = .31), or MP Col AUC (median, 58 U vs 73.5 U; *P* = .18), but sample size was not adequately powered to detect a difference. A significant difference was found between these groups in PFA-200 Col/ADP closure time (CT), with obstructed cats having higher median CT (median, 145 s vs 68 s; *P* = 0.02; Table 2).

**Table 1:** Summary of results of platelet function tests in cats with acute kidney injury (AKI) and control cats.

	Category	Median	SD	Minimum	Maximum
**MP ADP AUC (U)**	AKI	67	34.19	2	122
	Control	103	38.31	42	180
**MP ASPI AUC (U)**	AKI	18	13.33	5	46
	Control	42	19.86	23	85
**MP Col AUC (U)**	AKI	66	25.60	21	106
	Control	97	44.25	20	222
**PFA ADP/Col CT (s)**	AKI	82	50.24	50	208
	Control	51	15.26	41	105

Abbreviations: ADP = adenosine diphosphate; ASPI = arachidonic acid; AUC = area under the curve; Col = collagen; CT = closure time; MP = Multiplate®; PFA = platelet Function Analyzer.

**Table 2:** Summary of results of platelet function tests in cats with acute kidney injury (AKI) with and without ureteral obstruction as the cause.

	Category	Median	SD	Minimum	Maximum
**MP ADP AUC (U)**	Non-obstructed	64	28.44	26	106
	Obstructed	67	39.04	2	122
**MP ASPI AUC (U)**	Non-obstructed	25	14.77	12	46
	Obstructed	17	11.11	5	42
**MP Col AUC (U)**	Non-obstructed	73.5	22.7	41	106
	Obstructed	58	25.74	21	83
**PFA ADP/Col CT (s)**	Non-obstructed	68	19.7	50	103
	Obstructed	145.5	52.36	61	208

Abbreviations: ADP = adenosine diphosphate; ASPI = arachidonic acid; AUC = area under the curve; Col = collagen; CT = closure time; MP = Multiplate®; PFA = Platelet Function Analyzer.

Among cats with AKI, no association was found between serum urea, creatinine, or phosphorus concentrations, or urine specific gravity, and any platelet function test result (Table 3).

**Table 3:** *P*-values and correlation (*r*-value) for associations between renal and platelet function parameters by Pearson correlation analysis. No correlations were statistically significant.

		MP ADP AUC	MP AA AUC	MP Col AUC	PFA ADP/Col CT
**Creatinine**	Correlation	−0.38	0.06	−0.33	−0.07
	*P*-value	.21	.85	.27	.82
**Urea**	Correlation	−0.08	0.35	−0.14	0.07
	*P*-value	.8	.24	.66	.82
**USG**	Correlation	0.19	0.18	0.45	0.04
	*P*-value	.54	.55	.13	.90
**Phosphorus**	Correlation	−0.47	0.16	−0.03	−0.26
	*P*-value	.11	.61	.93	.38

Abbreviations: ADP = adenosine diphosphate; AUC = area under the curve; ASPI = arachidonic acid; Col = collagen; CT = closure time; MP = Multiplate®; PFA = platelet Function Analyzer; USG = urine specific gravity.

The previously described reference interval (RI) for PFA-200 ADP/Col CT in our laboratory is 19.5-87.2 s.^20^ Using these values, 8 cats with AKI (57%) were above the RI, and 5 cats (35%) had CT values higher than 120 s, the cut-off value for clopidogrel effect with this assay.^20^ No control group cats were outside of the RI.

Our laboratory RIs for ADP, Col, and ASPI using the MP analyzer are 11-176 AU, 32-129 AU and 59-129 AU, respectively.^22^ Using these values, no ADP results (0%), 3 Col results (20%), and 3 ASPI results (20%) in the AKI group had aggregation less than the RI. In the control group, 0%, 67%, and 6%, respectively, had values less than the RI. Given the lack of homogeneity in current and retrospective samples, statistical analysis was not performed to compare the number of samples outside of the reference interval.

## Discussion

Results of our study showed that cats with AKI have abnormalities in platelet function compared with control cats, and that these abnormalities may be more severe in cats with ureteral obstruction. These changes are a result of platelet dysfunction and not thrombocytopenia.

The physiologic effects of azotemia on coagulation may occur as a result of uremic toxins affecting the synthesis and function of coagulation factors and platelets; because of the loss or retention of proteins associated with coagulation and inflammation as a result of decreases in glomerular filtration rate and an ineffective glomerular filtration barrier; or as a result of the inflammatory and microvascular changes associated with damage to the kidneys.^1^^,^^6–8^ An acquired deficiency in vWF has been hypothesized to play a role in both dogs and humans.^2^^,^^10^ The cause of platelet dysfunction was not investigated in our study, but the changes seen with all MP agonists suggest that a deficiency in vWf is likely not the sole cause, because vWF deficiency is not expected to prolong impedance aggregometry with agonists other than ristocetin.^23^^,^^24^

Previous research on the effect of azotemia on feline platelet function is minimal. One study that investigated 6 cats with AKI using MP ASPI and MP ADP found increased platelet function in azotemic animals.^13^ The AUC values in control cats in that study were similar to ours. The discordance between these studies may be a consequence of sample size, severity of azotemia, or specific disease processes; for example, 4 of the 6 cats with AKI in the other study had known CKD.^13^ The previous study also noted significant differences in the rate of platelet clumping between groups, which was not the case in our study and may have influenced results. Lastly, the previous study used heparinized blood, whereas we used citrated samples.

We did not find a correlation between the severity of AKI and the severity of platelet dysfunction. It is possible that a larger sample size may have detected trends that were not seen in this group of cats, but it is also possible that once a threshold of azotemia occurs, the severity of platelet dysfunction does not progress. Although urea and creatinine are standard markers of renal dysfunction, they are not uremic toxins and are unlikely to directly affect platelets, and so other markers may correlate with platelet dysfunction.^12^

Although MP did not detect an association between platelet dysfunction and cause of azotemia, PFA-200 analysis showed more severe dysfunction in cats with ureteral obstruction. This result may be a consequence of the underlying obstructive process, or as a result of non-obstructed cats having inflammatory conditions such as pyelonephritis that increased platelet function and counteracted some of the azotemia-associated dysfunction. Different assays have different sensitives to various aspects of platelet function, and so it is possible that the changes present in obstructed cats may alter PFA results more than MP, for example, by altering platelet–membrane interactions. The differences in pathophysiology in obstructive disease compared with other causes of kidney injury, as well as possible genetic and environmental contributors to these conditions, may be the cause of the discordant changes between assays. It also may have been an artifact of small sample sizes, because the study was not adequately powered to detect a difference in subgroups. This observation may be clinically relevant, because cats with ureteral obstructions are likely to require surgical intervention and therefore have a higher risk of bleeding, however, further work in the area is needed to confirm this finding. One of 9 obstructed cats had a serum creatinine concentration within the reference interval, but would have been classified as grade II AKI by IRIS guidelines, as such this observation is unlikely to be a reason for these different results.

Our study did not investigate the occurrence or severity of bleeding in cats with AKI, and thus the clinical relevance of these findings is not known. Although cats in our study had not been treated with antithrombotics and thus a direct comparison to cats on treatment is not possible, the results suggest that a portion of azotemic cats have platelet dysfunction to an extent that may be comparable to those undergoing clopidogrel treatment.^20^^,^^25^ It is, however, unclear what degree of platelet dysfunction in cats on what tests correlates with an increased risk of clinical bleeding. Statistical analysis of this risk was not performed because it would include different groups of cats analyzed several years apart, with different reagent batches. Despite this, the presence of results of a magnitude comparable to antithrombotic drug effect suggests these changes in platelet function may be clinically relevant.

Our study had some limitations. Anemia has been documented to affect the results of platelet function tests,^26^ and a significantly lower hematocrit was present in the AKI group compared with the control cats. The effects of anemia on PFA-200 results in humans have been debated, with some studies showing no difference,^27^ and others suggesting a higher CT occurs with a lower hematocrit.^28^ The manufacturers of the PFA-200 state that hematocrits below 25% can increase CT^29^; 5 of the 15 cats in the AKI group had hematocrits less than 25%, and thus may have had prolonged CTs as a result. The lowest hematocrit in the AKI group was 21%, and no cat was severely anemic. No data are available on the effect of hematocrit on PFA-200 results in cats, and thus the effect of anemia in this population is unclear.

Likewise, some research has shown that anemia does not significantly affect the results of MP ADP and Col reagents, but may decrease the AUC for ASPI.^28^ Other research has shown no effect of anemia on MP AUCs.^26^ We suspect that the influence of anemia on platelet function in these cats was minor, however, if present would still be relevant to the clinical condition, because cats with AKI often demonstrate a component of anemia.^30^

One cat in our study had a MP AUC of 2 U, which is lower than would be expected in an animal with an adequate platelet count. This result may be spurious, and ideally would have been confirmed with repeat analysis. Unfortunately, the small sample sizes available precluded doing so. Other studies on platelet function in cats and dogs have reported near-zero values, suggesting that the occurrence of these low readings may be a common occurrence, and previously have been interpreted as physiologic.^13^^,^^31^^,^^32^

Concurrent medications also may affect platelet function. Although most of the cats in our study were receiving treatment, care was taken to exclude those receiving drugs known to be associated with thrombocytopathy. Nonetheless, many cats received treatments, most commonly antibiotics (primarily cefotaxime), opioids, and maropitant. The effects of these drugs on platelet function, when known, is likely minimal, but most data available are from humans and the effects in cats may not be the same.^33^^,^^34^ Fluid therapy also may affect platelet function. Although synthetic colloids were not administered to any cat, IV crystalloids may have caused dilution of blood components or interactions with the endothelial glycocalyx, leading to changes in platelet function. These possibilities represent the reality of evaluating animals with AKI presented to referral institutions, but indicate that the changes seen may not have been solely a consequence of azotemia.

Although a difference in platelet function between cats with and without AKI was statistically present, substantial overlap occurred between groups. It is also not clear what extent of platelet dysfunction correlates with increase in bleeding risk, and comparisons to antithrombotic treatment are not directly possible. As such, although cats with AKI have a biochemical tendency to platelet dysfunction, this tendency may not translate into clinical bleeding risk, and overlap with healthy cats is substantial. Although our study was adequately powered to detect a difference in cats with and without AKI, larger sample sizes would be needed to definitively determine if the cause or severity of azotemia is associated with the severity of platelet dysfunction. Further research in this area is required to determine the importance of these changes, the clinical relevance of platelet dysfunction in uremia, and possible strategies to mitigate this dysfunction.

### Conclusions

Cats with AKI have platelet dysfunction on PFA-200 ADP/Col testing and on MP testing in response to ADP, Col, and ASPI. The PFA-200 but not the MP identified more dysfunction in cats with ureteral obstruction. This observation is consistent with results in other species that exhibit platelet dysfunction in AKI. The clinical relevance of this dysfunction and treatment options require further investigation, but clinicians should be aware of the potential bleeding risk in cats with AKI, especially in those undergoing surgery.

## Supplementary Material

Supplementary_Material_aalag115

## Data Availability

The data that support the findings of this study are available on request from the corresponding author.

## Off-label antimicrobial declaration

Authors declare no off-label use of antimicrobials.

## Institutional animal care and use committee or other approval declaration

This study was approved by the University of Guelph Animal Care Committee, Animal Utilization Protocols 5262 and 4732.

## Appendix

### Inclusion criteria and criteria for categorization

Cats presenting to a university teaching hospital with a diagnosis of AKI were prospectively enrolled. AKI was diagnosed based on a combination of acute onset of clinical signs (decreased appetite, vomiting, lethargy, abdominal pain, increased or decreased urine output for less than 48 h before presentation), and either serum creatinine concentration above the laboratory reference interval (50-190 μmol/L) with decreased urine concentration (USG < 1.035); and ultrasonographic evidence of AKI (including renal pelvic or ureteral dilatation, peripheric steatitis, hyperechoic renal cortex); or pyelography consistent with ureteral obstruction. Pyelonephritis was diagnosed if a positive urine or pyelocentesis culture was obtained and no other cause of AKI was present.

Ureteral obstruction was diagnosed based on the clinical judgment of a attending radiologist and internal medicine specialist or criticalist based on clinical and imaging findings and exclusion of other causes. Imaging findings considered consistent with ureteral obstruction included abdominal ultrasonography demonstrating a renal pelvis diameter a minimum of >3 mm in the presence of concurrent hydroureter or obstructive ureterolithiasis; a renal pelvic diameter >13 mm; or obstruction noted on ultrasound-guided fluoroscopic anterograde pyelography; all performed by a board-certified radiologist.

Cats were included if they were diagnosed with AKI, if blood could be collected for platelet function testing via jugular venipuncture within 24 hours of admission to the hospital, and if owners consented to enrollment. For all patients a CBC, serum biochemistry panel, and urinalysis were required to be performed within 24 hours of platelet function analysis.

Cats were excluded if they were receiving drugs known or suspected to affect platelet function (antithrombotics, synthetic colloids, NSAIDs, sedatives other than gabapentin, trazodone, or opioids, or other drugs based on author discretion), had received general anesthesia within 24 h before blood collection, were positive for FeLV or FIV, or (in the control group) had anemia or (in both groups) thrombocytopenia or if there was a known history of CKD. Cats also were excluded if they were classified as IRIS Grade I (non-azotemic) AKI in order to assess only azotemic animals.

### Details of laboratory analysis

Samples were mixed with 15 full inversions. Slides were prepared from each EDTA sample and evaluated for platelet clumping by an investigator (MK). Samples were excluded if moderate to marked platelet clumping was noted, as defined in a prior publication as “Innumerable small, >2 moderate, or one large clump, platelet count subjectively low” or “None to rare free platelets, several large clumps.”^35^ Blood from the citrate tube was analyzed within 1 h of collection using the PFA-200 Col/ADP cartridge, and with MP using ADP, Col, and ASPI as agonists.

For PFA-200 analysis, samples were analyzed using the standard protocol for the laboratory, as previously described.^20^^,^^21^ CT was recorded.

For Multiplate® analysis, stock solutions of ADP (MP Biomedicals), ASPI (TCI America), and Col (Fisher Scientific) were prepared from bulk reagents and stored at in 200 μL aliquots at –80 °C for a maximum of 30 days, and once thawed stored at 4 °C for a maximum of 24 h. Test cells were warmed to 37 °C and the automated pipette used to draw 300 μL of 3 mM CaCl_2_ in 0.9% saline, followed by 300 μL of citrated whole blood; the sample then was incubated for 3 min, followed by addition of the agonist and duplicate analysis for 6 min. The final agonist concentrations used in each analysis were 6.5 μM ADP, 3.2 μg/mL Col, and 0.5 mM ASPI. AUC in aggregation U was recorded.

Sufficient sample was not available to allow repeat analysis of PFA and MP if errors occurred, and thus a “Flow Obstruction” error on PFA-200 or large CV on the MP resulted in the data point being discarded and a new cat enrolled. However, no cats were excluded for this reason over the course of the study.

## Abbreviations

ADP: adenosine diphosphate
ASPI: arachidonic acid
AUC: area under the curve
BMBT: buccal mucosal bleeding time
CKD: chronic kidney disease
Col: collagen
CT: closure time
MEA: multiple electrode aggregometry
PCT: plateletcrit
PFA-200: platelet function analyzer 200
PLT: platelet Count
U: multiplate aggregation units
