# Supplementary material for: Acute kidney injury is associated with decreased platelet function in domestic cats
Source: J Vet Intern Med. 2026 Jun 10;40(3):aalag115. doi: 10.1093/jvimsj/aalag115 (PMC13252594; doi:10.1093/jvimsj/aalag115)
Supplement: Supplementary_Material_aalag115 [file supplementary_material_aalag115.docx]

Supplementary Material

Control Group

| **PLT (k/μL)** | **MPV (fL)** | **PCT (%)** | **HCT (%)** | **WBC (x10^9)** |
| --- | --- | --- | --- | --- |
| 372 | 19.5 | 0.7254 | 39 | 6.9 |
| 332 | 17.7 | 0.58764 | 37 | 9.48 |
| 301 | 16.9 | 0.50869 | 37 | 10.8 |
| 207 | 12.9 | 0.26703 | 40 | 10.5 |
| 162 | 21 | 0.34 | 36 | 6.9 |
| 270 | 16.5 | 0.4455 | 34 | 7.0 |
| 444 | 14 | 0.6216 | 29 | 5.6 |
| 266 | 17.8 | 0.47348 | 35 | 9.3 |
| 371 | 11.3 | 0.41923 | 34 | 9.9 |
| 235 | 17.2 | 0.4042 | 47 | 7.2 |
| 302 | 13 | 0.3926 | 36 | 5.7 |
| 347 | 12.4 | 0.43028 | 37 | 12.8 |
| 266 | 17.8 | 0.47348 | 35 | 7.9 |
| 555 | 10.1 | 0.56055 | 33 | 7.2 |
| 422 | 14.8 | 0.62456 | 31 | 10.2 |

Acute Kidney Injury Group

| **PLT** | **MPV** | **PCT** | **HCT** | **WBC** |
| --- | --- | --- | --- | --- |
| 324 | 18 | 0.583 | 25 | 8.2 |
| 181 | 17.6 | 0.319 | 29 | 13.6 |
| 467 | 11.8 | 0.551 | 21 | 21.9 |
| 161 | 19.3 | 0.311 | 44 | 4.0 |
| 418 | 15.3 | 0.640 | 29 | 6.2 |
| 147 | 20.5 | 0.260 | 36 | 4.3 |
| 197 | 20 | 0.394 | 30 | 7.7 |
| 157 | 18.6 | 0.292 | 22 | 3.4 |
| 186 | 23.6 | 0.440 | 41 | 2.2 |
| 207 | 19.2 | 0.397 | 21 | 18.4 |
| 404 | 13.8 | 0.558 | 33 | 6.9 |
| 318 | 17.4 | 0.553 | 22 | 19.9 |
| 228 | 14.5 | 0.331 | 23 | 5.5 |
| 159 | 18.5 | 0.29415 | 25 | 12.3 |
| 188 | 15.3 | 0.288 | 23 | 10.1 |

Hematologic parameters from two groups of cats with and without Acute Kidney Injury (AKI) in a study of platelet function. Results are obtained from an ADVIA 2120i.PLT: Platelet Count HCT: Hematocrit; MPV: Mean Platelet Volume; PCT: Plateletcrit; WBC: White Blood Cell Count
